# Redefining β-blocker response in heart failure patients with sinus rhythm and atrial fibrillation: a machine learning cluster analysis

**DOI:** 10.1016/S0140-6736(21)01638-X

**Published:** 2021-10-16

**Authors:** Andreas Karwath, Karina V Bunting, Simrat K Gill, Otilia Tica, Samantha Pendleton, Furqan Aziz, Andrey D Barsky, Saisakul Chernbumroong, Jinming Duan, Alastair R Mobley, Victor Roth Cardoso, Karin Slater, John A Williams, Emma-Jane Bruce, Xiaoxia Wang, Marcus D Flather, Andrew J S Coats, Georgios V Gkoutos, Dipak Kotecha, card AIc group and the Beta-blockers in Heart Failure Collaborative Group

**Affiliations:** a University Hospitals Birmingham NHS Foundation Trust, Birmingham, UK; b Institute of Cancer and Genomic Sciences, University of Birmingham, Birmingham, UK; c Institute of Cardiovascular Sciences, University of Birmingham, Birmingham, UK; d Computer Sciences, University of Birmingham, Birmingham, UK; e Health Data Research UK Midlands Site, Birmingham, UK; f Norwich Medical School, University of East Anglia, Norwich, UK; g Warwick Medical School, University of Warwick, Warwick, UK

## Abstract

**Background:**

Mortality remains unacceptably high in patients with heart failure and reduced left ventricular ejection fraction (LVEF) despite advances in therapeutics. We hypothesised that a novel artificial intelligence approach could better assess multiple and higher-dimension interactions of comorbidities, and define clusters of β-blocker efficacy in patients with sinus rhythm and atrial fibrillation.

**Methods:**

Neural network-based variational autoencoders and hierarchical clustering were applied to pooled individual patient data from nine double-blind, randomised, placebo-controlled trials of β blockers. All-cause mortality during median 1·3 years of follow-up was assessed by intention to treat, stratified by electrocardiographic heart rhythm. The number of clusters and dimensions was determined objectively, with results validated using a leave-one-trial-out approach. This study was prospectively registered with ClinicalTrials.gov (NCT00832442) and the PROSPERO database of systematic reviews (CRD42014010012).

**Findings:**

15 659 patients with heart failure and LVEF of less than 50% were included, with median age 65 years (IQR 56–72) and LVEF 27% (IQR 21–33). 3708 (24%) patients were women. In sinus rhythm (n=12 822), most clusters demonstrated a consistent overall mortality benefit from β blockers, with odds ratios (ORs) ranging from 0·54 to 0·74. One cluster in sinus rhythm of older patients with less severe symptoms showed no significant efficacy (OR 0·86, 95% CI 0·67–1·10; p=0·22). In atrial fibrillation (n=2837), four of five clusters were consistent with the overall neutral effect of β blockers versus placebo (OR 0·92, 0·77–1·10; p=0·37). One cluster of younger atrial fibrillation patients at lower mortality risk but similar LVEF to average had a statistically significant reduction in mortality with β blockers (OR 0·57, 0·35–0·93; p=0·023). The robustness and consistency of clustering was confirmed for all models (p<0·0001 *vs* random), and cluster membership was externally validated across the nine independent trials.

**Interpretation:**

An artificial intelligence-based clustering approach was able to distinguish prognostic response from β blockers in patients with heart failure and reduced LVEF. This included patients in sinus rhythm with suboptimal efficacy, as well as a cluster of patients with atrial fibrillation where β blockers did reduce mortality.

**Funding:**

Medical Research Council, UK, and EU/EFPIA Innovative Medicines Initiative BigData@Heart.

## Introduction

Advances in therapeutics have substantially improved the prognosis of patients with heart failure with reduced ejection fraction (HFrEF). However, mortality remains unacceptably high (ie, greater than most cancers), especially in the majority of patients in clinical practice with multimorbidity—eg, atrial fibrillation, which is common in patients with HFrEF^1^, ^2^ and is associated with a considerably worse prognosis.^3^ In contrast to patients in sinus rhythm, β-adrenergic blockers, which are a cornerstone of heart failure treatment, were not shown to reduce mortality in patients with concomitant atrial fibrillation.^4^ Within the subgroup of patients with atrial fibrillation, conventional statistical analysis was unable to identify any single patient characteristic that determined efficacy in these patients.^4^

With prevalence of atrial fibrillation expected to double in the coming decades,^5^ better identification of patient subgroups that could benefit from therapy is critical to address this unsustainable burden on health-care services.^6^ Conversely, the ability to identify individuals who are unlikely to receive therapeutic benefit could allow for a more personalised medicine approach, by stratifying the use of additional management strategies available in clinical practice. This approach also applies to patients in sinus rhythm—although β blockers show 4% absolute risk reduction in mortality, the mortality rate in patients randomly assigned to β blockers was still 14% during 18 months of follow-up.^4^ Pipeline therapies in development could be used to target patients who are predicted to have a suboptimal response.^7^

**Research in context**

**Evidence before this study**

A search of PubMed (from database inception to May 25, 2021) using title or abstract terms “clustering” or “sub-group”, combined with title, abstract, or MeSH terms for “heart failure”, without language or other restrictions, identified 391 results (excluding duplicates). 19 of the studies identified were evaluated at a full-text level, 12 of which were related to clustering approaches in patients with heart failure. A wide variety of clustering methods was used in these studies, with a lack of transparency in methods, insufficient assessment of the robustness of cluster membership, and only one study with external validation.

**Added value of this study**

Using individual patient data from double-blind, randomised controlled trials, we determined clusters of patients with heart failure and reduced ejection fraction with differing prognostic response to β blockers. Identifying distinct clusters of patients, including in sinus rhythm with a lack of treatment response and in atrial fibrillation with significant mortality benefit from β blockers, this study provides a novel method to better classify patients with heart failure. The artificial intelligence pipeline developed is a model for future analysis across other medical conditions, with objective assessment and assignment to clusters, robust analysis of the consistency of clustering, and external cross validation.

**Implications of all the available evidence**

Artificial intelligence-based clustering approaches are able to incorporate and simultaneously adjust for multiple comorbidities. Their use could allow for more refined stratification of treatment response to common clinical treatments, leading to avoidance of adverse outcomes. Prospective evaluation in a randomised setting is now warranted to evaluate if these approaches are valuable in routine practice to improve patient prognosis, particularly in conditions such as heart failure, which remains a key driver of health-care cost and poor patient quality of life.

We hypothesised that artificial intelligence clustering techniques could establish distinct categories of patients corresponding to therapeutic efficacy, in individuals with sinus rhythm and atrial fibrillation. Unsupervised machine learning approaches have the potential to exceed preconceived associations, taking into account interactions beyond the capabilities of conventional statistical analysis.^8^, ^9^ We designed and tested a novel clustering approach in double-blind data from landmark randomised controlled trials (RCTs), with multilevel evaluation and validation to ensure robust and reproducible findings.

## Methods

### Study design

This study was performed by the card*AI*c group at the University of Birmingham and University Hospital Birmingham NHS Foundation Trust, UK, using individual patient data (IPD) from double-blind, placebo-controlled RCTs collated by the Beta-blockers in Heart Failure Collaborative Group (BB-meta-HF).

This international academic consortium was established to bring together investigators and pharmaceutical companies to address questions about the therapeutic efficacy of β blockers, and important clinical comorbidities in heart failure.^10^, ^11^, ^12^, ^13^ In brief, RCTs were eligible for inclusion if they recruited more than 300 patients, were not confounded by investigation of other treatments, had a planned follow-up of more than 6 months, and explicitly reported mortality as an endpoint.^10^ The planned studies by BB-meta-HF were prospectively registered with ClinicalTrials.gov (NCT00832442) and the PROSPERO database of systematic reviews (CRD42014010012).^14^ Nine RCTs of β blockers versus placebo which assessed patients in sinus rhythm and atrial fibrillation were included. The full list of included and excluded trials is presented in the appendix (p 7). Using the Cochrane Collaborations Risk of Bias Tool, we established that each trial had low risk of bias.^15^ All included studies had appropriate ethical approval.

A standardised data request form to obtain IPD from each trial has been published, along with search results and individual study demographics.^10^ IPD were extracted from original source files and all data were cross-checked and compared with published reports. Discrepancies, inconsistencies, and incomplete data were checked against original case report forms and trial documentation to ensure IPD integrity. Trial databases were then harmonised according to the standardised data approach to match patient characteristics and outcomes across all trials.

### Population and data

Participants with left ventricular ejection fraction (LVEF) of 50% or more were excluded due to previous analysis demonstrating a lack of efficacy from β blockers regardless of rhythm.^12^ The remainder were stratified by heart rhythm on the baseline electrocardiogram (ECG) into sinus rhythm or atrial fibrillation. Atrial flutter was included in the atrial fibrillation group, but accounted for only 4%.^4^ Individuals with a missing ECG or paced rhythm were excluded. To identify meaningful phenotypic subgroups, we prespecified a list of common variables available at baseline that are known to be relevant to outcomes in patients with HFrEF; namely, age, gender, body-mass index, heart rate, systolic blood pressure, LVEF, previous myocardial infarction, New York Heart Association class (I or II *vs* III or IV), creatinine, and baseline drug therapy (angiotensin-converting enzyme inhibitor, angiotensin receptor blocker, diuretics, anticoagulants and digoxin). To avoid introducing bias for data not missing completely at random, only participants with complete case data for all variables were used to establish the clustering subsets.

Variational autoencoders (VAEs), a neural network-based approach, were used for dimensionality reduction. VAEs can cater for heterogeneous data types, including numerical and nominal variables using different likelihood models (appendix pp 2, 13). Compared with conventional principal component analysis (PCA), the VAE method allows for discovery of complex non-linear relationships, and was significantly better in this dataset (average best gap statistic value 0·812 for PCA *vs* 1·447 for VAE; p=0·0001).

### Outcome

The outcome for this analysis was all-cause mortality, which included additional deaths reported after the censor date for a number of included studies.^4^ Each trial performed independent adjudication of clinical outcomes. Assignment of outcomes in this study was based on an intention-to-treat approach according to the randomised group, regardless of treatment discontinuation or crossover. There were no patients with missing vital status.

### Clustering

**Figure 1:**
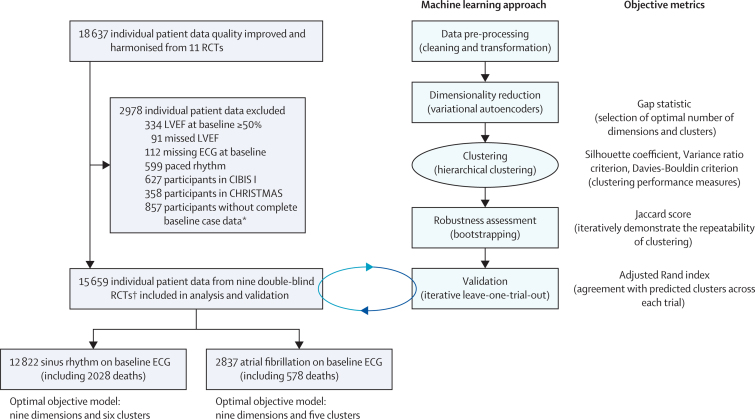
Study flowchart ECG=electrocardiogram. LVEF=left ventricular ejection fraction. RCTs=randomised controlled trials. *Age, gender, body-mass index (missing n=148), heart rate (missing n=5), systolic blood pressure (missing n=7), LVEF, previous myocardial infarction (missing n=43), New York Heart Association symptom class (missing n=83), creatinine (missing n=101), baseline drug therapy (angiotensin converting enzyme inhibitor or angiotensin receptor blocker, diuretics, anticoagulants [missing n=1] and digoxin), and other than sinus rhythm or atrial fibrillation or flutter on baseline ECG (n=506). 32 patients had more than one variable missing. † MDC, US-HF, ANZ, CIBIS-II, MERIT-HF, COPERNICUS, CAPRICORN, BEST, and SENIORS (appendix p 16).

The aim of the cluster analysis was to find similar subgroups within the cluster, yet dissimilar to other clusters (minimising intercluster similarity). Hierarchical clustering (iteratively merging samples to form groups) and k-means++ (iteratively assigning samples to cluster centres) were used as the underlying clustering techniques. The number of clusters and the number of dimensions used were objectively established by iteratively repeating clustering and then selecting the model with the highest gap statistic value, followed by evaluation with three performance measures (figure 1; appendix pp 3–5, 14). The gap statistic measures the result of the clustering of the data versus a random model using the same number of clusters. The higher the gap value, the less likely the identified clusters were assigned at random. Radar plots were used to visually describe the generated clusters for key demographic characteristics. The plots were generated by scaling or normalising (or both) the median of that specific cluster to the median of the complete cohort, or mean percentage for categorical variables.

### Evaluation and validation protocol

Clustering was further evaluated for robustness and validation. The robustness of the approach was confirmed using repeated clustering (*k*=100) of random subsets of the data (bootstrapping), comparing the resulting clustering against random cluster assignments.^16^ The overlap of clusters was identified using the weighted Jaccard score, and the comparison to random assignment assessed using the Kolmogorov–Smirnov test. Validation of the overall approach was shown by repeatedly generating clusters, with each iteration leaving one trial out. Because each trial is unique, with its own selection criteria, this approach provided external validation of clustering results. The Adjusted Rand Index (ARI) was used to measure the agreement between the clustering approaches compared with a random model. Further details on the clustering and evaluation approaches used are presented in the appendix (pp 2–6).

### Statistical analysis

A statistical analysis plan was generated and finalised in advance of data analysis. Summary results are presented as percentages, or median (IQR). Group comparisons were made using the Kruskal-Wallis non-parametric rank test. We calculated the odds ratios (ORs) and risk ratios for each cluster assignment by splitting each cluster into placebo and β blocker treatment and accounting for all sources of death. The number needed to treat (NNT) was calculated as the inverse of the absolute risk reduction (ARR) comparing β blockers with placebo. A two-tailed p value of 0·05 was considered statistically significant. Analyses were performed using the Python library statsmodel (version 0.12.1) on Python (version 3.7.2), and Stata (version 14.2).

### Role of the funding source

The funders of the study had no role in study design, data collection, data analysis, data interpretation, or writing of the report.

## Results

From the nine trials, 15 659 patients with HFrEF were included, with median age of 64 years (IQR 55–72); 3708 (24%) of the patients were women. Median LVEF was 27% (IQR 21–33), with the majority of patients reporting severe or disabling symptoms (table 1). According to their baseline ECG, 12 822 patients were in sinus rhythm and 2837 patients were in atrial fibrillation (figure 1). Individuals with atrial fibrillation were older with lower rates of previous myocardial infarction, but similar heart rate, blood pressure, and LVEF compared with patients in sinus rhythm. Due to the randomisation in each trial, there were no differences in patient characteristics between β blockers or placebo for either sinus rhythm or atrial fibrillation (appendix p 8). The median follow-up period was 1·3 years (IQR 0·9–1·9).

**Table 1:** Baseline characteristics

		**All patients (n=15 659)**	**Sinus rhythm (n=12 822)**	**Atrial fibrillation (n=2837)**
Age, years		64 (55–72)	64 (54–71)	69 (60–74)
Sex				
	Women	3708 (23·7%)	3185 (24·8%)	523 (18·4%)
	Men	11 951 (76·3%)	9637 (75·2%)	2314 (81·6%)
Body-mass index, kg/m^2^		26·6 (24·0–29·8)	26·6 (24·0–29·7)	26·9 (24·3–30·1)
Heart rate, beats per min		80 (72–88)	80 (72–88)	81 (72–92)
Systolic blood pressure, mm Hg		124 (110–140)	123 (110–139)	126 (113–140)
LVEF		27% (21–33)	27% (21–33)	27% (21–33)
Previous myocardial infarction		8538 (54·5%)	7411 (57·8%)	1127 (39·7%)
NYHA class III or IV		8802 (63·7%)	7048 (61·9%)	1754 (72·6%)
Creatinine, μmol/L		105 (88–124)	104 (88–124)	108 (90–131)
ACEi or ARB		14 877 (95·0%)	12 188 (95·1%)	2689 (94·8%)
Any diuretic therapy		13 563 (86·6%)	10 914 (85·1%)	2649 (93·4%)
Anticoagulation therapy		5033 (32·1%)	3379 (26·4%)	1654 (58·3%)
Digoxin		9299 (59·4%)	6919 (54·0%)	2380 (83·9%)

Data are median (IQR) or n (%). Breakdown according to randomised treatment allocation (β blockers *vs* placebo) is provided in the appendix (p 8). ACEi=angiotensin converting enzyme inhibitor. ARB=angiotensin receptor blocker. LVEF=left ventricular ejection fraction. NYHA=New York Heart Association.

Across the whole cohort of patients in sinus rhythm (n=12 822), β blockers significantly reduced all-cause mortality compared with placebo, with an adjusted OR of 0·74 (95% CI 0·67–0·81; p<0·001). Of the patients randomly assigned to β blockers, 907 (13·9%) of 6546 died, compared with 1121 (17·9%) of 6276 allocated to placebo.

**Figure 2:**
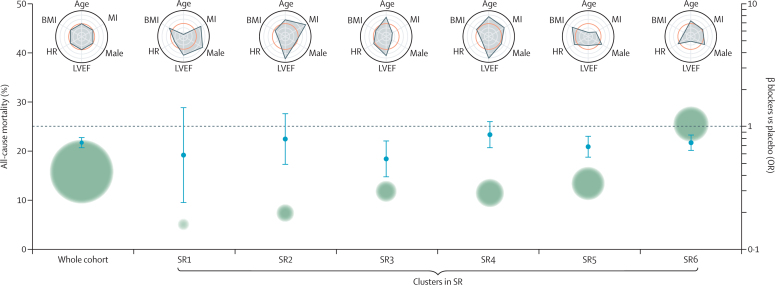
Clustering for all-cause mortality and β-blocker efficacy in SR Green circles represent the average mortality risk, with size relative to the number of patients in that cluster. ORs (95% CI) are for the efficacy of β blockers versus placebo for all-cause mortality; odds below the dotted line indicate a benefit from β blockers. Radar plots summarise scaled variables for each cluster, with the average for the whole cohort of sinus rhythm patients noted in orange. Values closer to the outer ring are higher than the cohort average for each of the key variables. Other variables not displayed in the radar plots include: systolic blood pressure, New York Heart Association symptom class, creatinine, and baseline drug therapy (appendix p 9). OR=odds ratio. BMI=body-mass index. HR=heart rate. LVEF=left ventricular ejection fraction. MI=myocardial infarction. SR=sinus rhythm.

The optimal VAE clustering for the primary analysis model in sinus rhythm used nine dimensions and six clusters (SR1–SR6). The majority of clusters showed a consistent benefit from β blockers on mortality across the risk of death, ranging from OR of 0·54 to 0·74 (table 2; figure 2). This included the two largest clusters, cluster SR5 encompassing younger patients with predominantly non-ischaemic cause (NNT 22·9), and SR6 including patients with the lowest LVEF and highest annualised death rate of 19·6% (NNT 17·4). The OR was stable in the smallest clusters—eg, SR1 at the lower end of mortality risk (annualised 3·9%), although the small sample size precluded statistical significance. Despite a large sample size (n=2537) and mid-range mortality risk (annualised 8·8%), cluster SR4 demonstrated no significant efficacy (OR 0·86, 95% CI 0·67–1·10; p=0·22), with cluster interrogation suggesting older patients with less severe symptoms and lower heart rate than average (appendix p 9). We evaluated the distribution of individual patient data and found a broad representation of trials across each cluster. Additional models were consistent regardless of the number of clusters (appendix p 15).

**Table 2:** Cluster-specific results for all-cause mortality

	**Annualised mortality**	**Placebo**	**β blockers**	**Odds ratio (95% CI)**	**Risk ratio (95% CI)**	**p value**	**Number needed to treat (95% CI)**
**SR**							
SR all	15·8%	1121/6276 (17·9%)	907/6546 (13·9%)	0·74 (0·67–0·81)	0·86 (0·81–0·90)	<0·0001	25 (18–39)
SR1	3·9%	14/222 (6·3%)	8/211 (3·8%)	0·59 (0·24–1·43)	0·74 (0·41–1·29)	0·23	NA
SR2	5·7%	40/487 (8·2%)	34/514 (6·6%)	0·79 (0·49–1·27)	0·89 (0·69–1·14)	0·33	NA
SR3	9·1%	108/731 (14·8%)	59/683 (8·6%)	0·54 (0·39–0·76)	0·71 (0·57–0·87)	0·0004	16 (11–36)
SR4	8·8%	151/1231 (12·3%)	140/1306 (10·7%)	0·86 (0·67–1·10)	0·93 (0·82–1·05)	0·22	NA
SR5	10·3%	267/1706 (15·7%)	202/1791 (11·3%)	0·69 (0·56–0·83)	0·82 (0·74–0·92)	0·0001	23 (15–47)
SR6	19·6%	541/1899 (28·5%)	464/2041 (22·7%)	0·74 (0·64–0·85)	0·86 (0·80–0·93)	<0·0001	17 (12–33)
**AF**							
AF all	20·4%	300/1425 (21·1%)	278/1412 (19·7%)	0·92 (0·77–1·10)	0·96 (0·87–1·05)	0·37	NA
AF1	13·8%	50/307 (16·3%)	59/301 (19·6%)	1·25 (0·83–1·90)	1·12 (0·92–1·36)	0·29	NA
AF2	9·2%	50/338 (14·8%)	29/321 (9·0%)	0·57 (0·35–0·93)	0·73 (0·54–0·98)	0·023	17 (9–119)
AF3	15·1%	68/348 (19.5%)	69/348 (19·8%)	1·02 (0·70–1·48)	1·00 (0·84–1·22)	0·92	NA
AF4	28·4%	81/201 (40·3%)	68/202 (33·7%)	0·75 (0·50–1·13)	0·87 (0·70–1·07)	0·17	NA
AF5	17·0%	51/231 (22·1%)	53/240 (22·1%)	1·00 (0·65–1·55)	1·00 (0·81–1·24)	1·0	NA

Data are % or n/N (%), unless stated otherwise. Results are based on objective assessment for the number of dimensions and clusters for sinus and atrial fibrillation, as defined by the gap statistic. NA=not applicable as the absolute risk reduction with β blockers is not significant. SR=sinus rhythm. AF=atrial fibrillation.

Robustness of clustering in sinus rhythm was confirmed, with good repeatability of the clustering approach using bootstrap methods (mean Jaccard score 0·575 [SD 0·103], compared with 0·121 [SD 0·005] with random assignment; p<0·0001). The validity protocol demonstrated consistency of cluster membership prediction in the leave-one-study-out approach (average ARI of 0·493 [SD 0·092]; appendix p 10) and for the predicted cluster membership in the left-out trials (average ARI 0·569 [SD 0·052]), which were significantly better than random cluster assignment (p=0·0198).

Across the whole cohort of patients in atrial fibrillation (n=2837), β blockers did not significantly reduce all-cause mortality compared with placebo, with an adjusted OR of 0·92 (95% CI 0·77–1·10; p=0·37). In participants randomly assigned to β blockers, 278 (19·7%) of 1412 died, compared with 300 (21·1%) of 1425 allocated to placebo.

**Figure 3:**
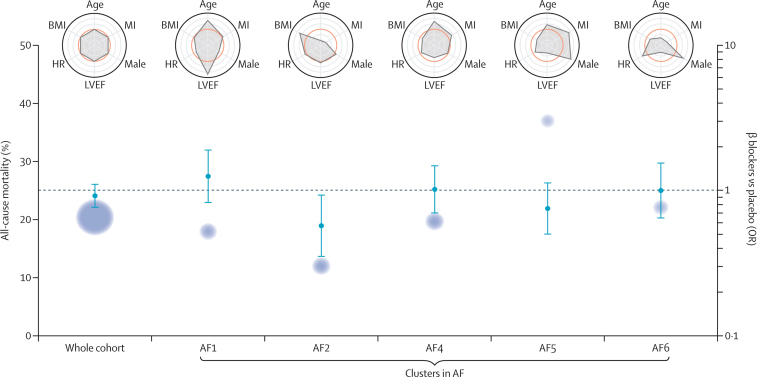
Clustering for all-cause mortality and β-blocker efficacy in AF Blue circles represent the average mortality risk, with size relative to the number of patients in that cluster. ORs (95% CI) are for the efficacy of β blockers versus placebo for all-cause mortality; odds below the dotted line indicate a benefit from β blockers. Radar plots summarise scaled variables for each cluster, with the average for the whole cohort of atrial fibrillation noted in orange. Values closer to the outer ring are higher than the cohort average for each of the key variables. Other variables not displayed in the radar plots include: systolic blood pressure, New York Heart Association symptom class, creatinine, and baseline drug therapy (appendix p 11). OR=odds ratio. AF=atrial fibrillation. BMI=body-mass index. HR=heart rate. LVEF=left ventricular ejection fraction. MI=myocardial infarction.

The optimal VAE clustering in atrial fibrillation used nine dimensions and five clusters (AF1–AF5). Consistent with the overall efficacy results, clusters AF1, AF3, and AF5 demonstrated no significant reduction in mortality with β blockers (table 2; figure 3). A lack of efficacy was also noted in cluster AF4, comprised of patients with low LVEF and markedly elevated mortality risk (annualised 28·4%). Compared with placebo, there was a statistically significant reduction in mortality with β blockers in cluster AF2, with an adjusted OR of 0·57 (95% CI 0·35–0·93; p=0·023) and NNT of 17·4. This cluster (n=659) was comprised of younger patients with lower rates of previous myocardial infarction, but similar LVEF to the average atrial fibrillation patient (appendix p 11). Although still elevated compared with most sinus rhythm patients, annualised mortality was the lowest of any atrial fibrillation cluster (9·2%).

Robustness of cluster membership in atrial fibrillation was confirmed, with a mean Jaccard score of 0·571 (SD 0·073; p<0·0001 *vs* random). The leave-one-study-out protocol confirmed validity (appendix p 12), with consistent ARI values for the iteration set (average 0·551 [SD 0·156]) and for the left-out study prediction (average 0·532 [SD 0·276]), which were significantly different to random (p=0·0264).

## Discussion

Application of a novel machine learning approach within RCTs was able to define clusters of treatment response for β blockers in patients with HFrEF. In sinus rhythm, specific clusters were identified where β blockers had suboptimal impact on mortality. Conversely, despite an overall lack of efficacy from β blockers in individuals with atrial fibrillation, this approach was able to identify a subgroup of atrial fibrillation patients where β blockers did reduce death. Clustering was based on objective assessment, and the robustness and validation protocols provide reassurance of the consistency and repeatability of findings. Development and application of these processes could facilitate an individualised approach to treatment selection that accounts for comorbidities, thereby contributing to improvements in patient wellbeing.

Heart failure continues to present an enormous and costly challenge for health-care services. Patients with heart failure are increasingly multimorbid, with the majority now with three or more comorbidities.^17^ Non-cardiac comorbidities can have a different clinical impact on heart failure, both for adverse events^18^ and patient-related health outcomes.^19^ Multimorbidity also complicates the management of patients with heart failure and adds to the challenge of identifying who, in routine practice, will benefit from recommended medication, or should be considered or referred for alternative therapy. Although the prevalence of comorbidities in HFrEF trials has increased over time, so have exclusion criteria for chronic kidney disease, anaemia, atrial fibrillation, and chronic lung and liver disease,^20^ making it more difficult to apply evidence to individual patients. Tools that can aid clinicians to stratify potential treatment responses are urgently needed to curtail the burden of heart failure on health and social care.^21^ There is also an opportunity to facilitate new clinical research and identify new drug targets. Atrial fibrillation is a prime example due to the excess morbidity and mortality when associated with HFrEF, and the lack of supporting evidence for many of the therapies currently recommended in clinical guidelines.^22^, ^23^

In this study, we developed and used a novel combination of machine learning methods to demonstrate the value of assessing the interaction of factors related to β-blocker efficacy in higher dimensions, which is not possible with conventional statistical analysis. Our approach started with the use of neural network-based autoencoders to isolate key features in the data, while capturing the latent and underlying structure of complex data interactions without reliance on preconceived hypotheses. Objective selection of the number of dimensions and clusters was done, which in previous studies is often at the discretion of researchers and hence subject to considerable (and unquantifiable) bias. Iterative hierarchical clustering was then tested by a thorough evaluation of robustness and consistency, in addition to repeated external validation using a leave-one-trial-out approach. Our rationale was to ensure confidence in the results of clustering for β-blocker efficacy, addressing often stated limitations in the value of clustering methods. Unlike previous studies that have clustered data from observational cohorts,^24^ we used the highest quality data obtained and harmonised from landmark, placebo-controlled RCTs with adjudicated endpoints.

Although tested in trials of β blockers, these approaches based on simple parameters have clear potential across the spectrum of therapies in heart failure, and across other cardiovascular and non-cardiovascular conditions. In sinus rhythm, we identified clusters of patients with reduced mortality benefit from β-blocker therapy, a clinically important finding that could allow targeting of alternative therapies to address the high residual rate of adverse outcomes in patients with HFrEF (despite optimal medical treatment). Notably for the lack of efficacy in the cluster with less severe symptoms, higher age, and lower heart rate; the combination of which would be consistent with the presumed mode of action of β blockers. The efficacies of different β blockers are known to depend on the disease substrate within each patient,^25^ in addition to genetic factors such as polymorphisms of the β1-adrenergic receptor.^26^ In atrial fibrillation, a cluster of patients was identified with a therapeutic response to β blockers—a significant reduction in mortality that was distinct from other clusters and the group as a whole. The potential gain from using these artificial intelligence approaches in clinical practice would be to isolate these patients and personalise their management, improving prognosis for this group, as well as avoiding adverse events from β blockers in those unlikely to receive prognostic benefit.

Approaches based on artificial intelligence are often criticised for their lack of transparency (black-box effect), although the point of these methods is often to go beyond conventional preconceptions of how data points are correlated. This does mean that translation to linear factor combinations is not possible. However, we have attempted to provide some underlying interrogation of the clustering approach by demonstrating how patients within each cluster differ from each other across some key variables. The radar plots confirm our hypothesis about the value of higher-level interactions of clinical factors—eg, when comparing the sinus rhythm cluster SR3 with SR4 (different β-blocker effectiveness) and SR6 (markedly different mortality rate). In atrial fibrillation, the cluster demonstrating benefit from β blockers primarily consisted of younger patients at lower overall mortality risk. This result could be consistent with patients with a less severe atrial fibrillation phenotype,^27^ and before the onset of multimorbidity that is critical to adverse outcomes in atrial fibrillation.^28^ Other advanced bioinformatic techniques in heart failure have used latent class analysis to define distinct comorbidity clusters with respect to heart failure rehospitalisation and mortality,^29^ and neural networks to predict incident heart failure in electronic health records.^30^ We are not aware of any previous approaches that have used such extensive validation of the assignment to clusters, reassignment to clusters, and also the predicted effects across different datasets.

As with any machine learning clustering approach, some degree of randomness is inevitably introduced during the embedding and dimensionality reduction steps. This is a desired approach to avoid suboptimal embeddings, and repeatability of findings was shown in the leave-one-trial-out validation protocol. Although the trial data cover a period before the routine use of cardiac resynchronisation therapy, neprilysin inhibition, and more recently gliflozins, the evidence base for all of these heart failure therapies is based on pre-existing renin-angiotensin and β1 adrenoreceptor blockade. Our study is limited to the outcome of mortality to avoid confounding by indication with hospitalisation and competing risk; further work is in progress to develop novel machine learning methodology. A particular strength of our data is the use of individual patient-level data from landmark RCTs that were carefully harmonised and quality controlled before analysis. The heart rhythm for each patient was established using their baseline ECG; while this is a more robust way to ascertain atrial fibrillation than clinical history, we might have missed patients with paroxysmal atrial fibrillation and had no information on the duration or burden of atrial fibrillation. Finally, this cohort consists of patients with HFrEF (primarily at the more severe end) and so the results might not apply to atrial fibrillation patients without a clinical diagnosis of heart failure.

This study has demonstrated the potential clinical value of combining a series of robust artificial intelligence-based approaches to better identify clusters of treatment response for a fundamental therapy used in patients with heart failure. The unsupervised and validated approach was able to consistently identify patient subgroups in sinus rhythm with suboptimal efficacy from β blockers, and importantly a subgroup of patients with atrial fibrillation in whom β blockers significantly reduced the risk of death despite an overall neutral response. The results warrant further validation across other treatments and conditions, followed by prospective evaluation to confirm if using such methods to direct the choice of therapy can improve patient outcomes.

## Data sharing

Individual patient data from the Beta-blockers in Heart Failure Collaborative Group are not available for sharing due to contractual requirements from the source data owners. The card*AI*c group are committed to open-source publication of algorithms and machine learning innovations (appendix p 2).

## Declaration of interests
