# Supplementary material for: Redefining β-blocker response in heart failure patients with sinus rhythm and atrial fibrillation: a machine learning cluster analysis
Source: Lancet. 2021 Oct 16;398(10309):1427–35. doi: 10.1016/S0140-6736(21)01638-X (PMC8542730; doi:10.1016/S0140-6736(21)01638-X)
Supplement: Supplementary appendix [file mmc1.pdf]

THE LANCET

Supplementary appendix

This appendix formed part of the original submission and has been peer reviewed. We post it as supplied by the authors.

Supplement to: Karwath A, Bunting KV, Gill SK, et al. Redefining β-blocker response in heart failure patients with sinus rhythm and atrial fibrillation: a machine learning cluster analysis. *Lancet* 2021; published online Aug 30. http://dx.doi.org/10.1016/S0140-6736(21)01638-X.

Redefining beta-blocker response in heart failure patients with sinus rhythm and atrial fibrillation: card*AI*c-cluster

The Lancet 2021; DOI: 10.1016/S0140-6736(21)01638-X

Supplementary appendix

SUPPLEMENTARY METHODS 2
(Variational) Autoencoders 2
Clustering analysis and techniques 2
Cluster performance measures 3
Silhouette coefficient 3
Variance ratio criterion 4
Davies-Bouldin criterion 4
Gap statistic 5
Evaluation 5
Jaccard Index 5
Adjusted Rand Index 5
Validation protocol 6

Supplementary Table 1: Included and excluded trials 7
Supplementary Table 2: Baseline characteristics according to randomised treatment allocation 8
Supplementary Table 3: Cluster characteristics in sinus rhythm 9
Supplementary Table 4: Validation protocol in the sinus rhythm cohort 10
Supplementary Table 5: Cluster characteristics in atrial fibrillation 11
Supplementary Table 6: Validation protocol in the atrial fibrillation cohort 12

Supplementary Figure 1: Simplified overview comparing neural networks with autoencoders 13
Supplementary Figure 2: Cluster selection based on the gap statistic 14
Supplementary Figure 3: Eight-cluster model for beta-blocker efficacy in sinus rhythm 15

REFERENCES 16

SUPPLEMENTARY METHODS

(Variational) Autoencoders

Autoencoders (or encoder-decoders) are an artificial neural network model trained to learn an efficient encoding of the input data of observations.[1] The designs of various approaches differ in detail, but nearly all model the observational input as input layers using the same dimensionality as the output layer. Between the input and output layers are one or more layers, each reduced in size. Autoencoders are commonly designed to be symmetric, with a reduced number of neurons in the most central layer. In the simplest version, an autoencoder would consist of three layers: one input layer, one hidden layer and one output layer. The hidden layer would have, potentially substantially, less neurons than the input and output layers and the output layer would have the same number of neurons as the input layer. Given input vectors for each observation, the model is than trained to learn the identity function for any input vector. As the hidden layer has essentially a lower dimension than both input and output, working as a data information bottleneck, any model that can reconstruct the original input from this reduced middle layer does encode the essence of the respective input observations. See **Supplementary Figure 1** for a schematic comparison between normal neural networks and autoencoders.

Autoencoders have been used in a variety of applications as dimensionality reduction (or embedding) schemes[2], as they are able to capture non-linear dependencies of the data. Depending on the size of the dataset, they can also be more efficient to compute. However, as an autoencoder model is a neural network, the initialization as well as the batch learning are to some degree random. Hence, each training of such a model might result in a different encoding, whereas conventional principal component analysis (PCA) should result in the same output at each run. Autoencoders are generally assumed to be able to learn more powerful generalizations when compared to PCA.

One particular type of autoencoder is the variational autoencoder (VAE). These are deep generative models that are highly expressive and are able to the capture the latent and underlying structure of potentially complex data. In contrast to normal autoencoders, they can also generate new data points (observations). Common approaches use the trained autoencoder model to generate novel data. A recent development employs the generative capabilities of VAEs to also generate missing data during training [3]. The Heterogeneous-Incomplete (HI)-VAE approach used in this study is specifically designed to cater for heterogeneous data, such as different kind of numerical variables (discrete count data, positive real-valued data, and real-valued data) and nominal variables (categorical and ordinal data). In detail, the likelihood models employed are as follows:

Numerical variables:

- Real-valued data, which takes values in the real line, i.e., $x_{nd} \in R$ uses a Gaussian likelihood model, i.e., $p(x_{nd}|\gamma_{nd}) = \mathcal{N}\left(x_{nd}|\mu_d(z_n), \sigma_d^2(z_n)\right)$
- Positive real-valued data, which takes values in the positive real line, i.e., $x_{nd} \in R^+$ uses a log-normal likelihood model, i.e., $p(x_{nd}|\gamma_{nd}) = log\ \mathcal{N}\left(x_{nd}|\mu_d(z_n), \sigma_d^2(z_n)\right)$
- (Discrete) count data, which takes values in the natural numbers, i.e., $x_{nd} \in \{1, \cdots, \infty\}$ is modelled using a Poisson likelihood model, i.e., $p(x_{nd}|\gamma_{nd}) = Poiss\left(x_{nd}|\lambda_d(z_n)\right)$

Nominal variables:

- Categorical data, which takes values in a finite unordered set, e.g., $x_{nd} \in \{\text{‘}blue\text{’}, \text{‘}red\text{’}, \text{‘}black\text{’}\}$. Assumes a multinomial logit model such that the R-dimensional output of a deep neural network $\gamma_{nd} = \{h_{d0}(z_n), h_{d1}(z_n), \cdots, h_{d(R-1)}(z_n)\}$ represents the vector of unnormalized probabilities, such that the probability of every category is given by $p(x_{nd} = r|\gamma_{nd}) = \frac{exp^{-h_{dr}(z_n)}}{\sum_{q=1}^{R} exp^{-h_{dq}(z_n)}}$
- Ordinal data, which takes values in a finite ordered set, e.g., $x_{nd} \in \{\text{‘}never\text{’}, \text{‘}sometimes\text{’}, \text{‘}often\text{’}, \text{‘}usually\text{’}, \text{‘}always\text{’}\}$ and where the probability of each (ordinal) category can be computed as $p(x_{nd} = r|\gamma_{nd}) = p(x_{nd} \le r|\gamma_{nd}) - p(x_{nd} \le r-1|\gamma_{nd})$ with $(x_{nd} \le r|z_n) = \frac{1}{1+exp^{-(\theta_r(z_n)-h_d(z_n))}}$.

We have made the VAE implementation available via PyPi.org (https://pypi.org/project/hivae/).

Clustering analysis and techniques

Cluster analysis, or clustering for short, is an approach originating from unsupervised machine learning (ML), by which the ML algorithm groups similar samples together based on some form of similarity or distance measure. A variety of clustering techniques and methodologies exist. Probably the most known and commonly used representatives are

hierarchical clustering and the partition-based algorithm *k*-means. Other approaches like DBSCAN[4] are based on finding clusters based on density, or use artificial neural networks to find similar sub-groups.[5]

Hierarchical clustering can be divided loosely into two main approaches: 'Top-down', where all observations initially form a single cluster and are subsequently divided into smaller partitions according to one or more features; and 'Bottom-up', where each individual observations are considered a single cluster (singleton) and the most similar clusters are merged incrementally.

The most common example of a bottom-up approach is agglomerative clustering. To measure the (dis)similarity between sets of observations, a metric is required. Common metrics for numerical observations include the Euclidian distance, the Manhattan distance, and the Mahalanobis distance. For other data types, such as categorical data, text observations or binary vectors, other metrics can be applied. The calculation of distance or similarity between sets of observations is not only based on the employed metric, but also by the linkage type used. Broadly speaking, the linkage criterion, which defines how to calculate the similarity between two clusters is based on the two closest observations of the two clusters (single-linkage), the farthest observations (complete linkage), (weighted) average of all observations, or based on the distances of the cluster centres (centroids). The agglomerative hierarchical clustering algorithm merges successive clusters until only one single cluster exists. During this iterative merging, each two clusters are merged based on a specific distance. By doing so, it produces a history of mergers, which can also be efficiently stored and viewed in the form of a tree-based dendrogram. This dendrogram can be used define clusters by choosing different cut-off points in the tree; a high cut-off results in lower number of clusters while a lower cut-off results in more clusters. In the extreme case, a distance of 0.0 between clusters results in the complete set of observations.

Whereas agglomerative clustering merges start from individual examples, *k*-means employs a different partitioning approach. The basic *k*-means algorithm initially assigns *k* observations to be cluster centres or centroids, and then assigns each observation to its nearest centroid. Once, all observations are assigned to one of the *k* clusters, the new centres for each cluster are calculated based on the high-dimensional mean of the observations. This process is repeated either until the cluster assignment converges or a maximum number of iterations has been reached. Similar to hierarchical clustering, the cluster analysis resulting from *k*-means depends to a large extend on the employed distance measure and all of the above-mentioned distance measures can be employed within the scope of the algorithm. Variations of the general *k*-means approach exist, most notably the *k*-medians algorithm and *k*-medoids algorithm. The first simply replaces the mean in each dimension during calculation of new cluster centres with the median in each dimension, while the latter (*k*-medoids or partitioning around medoids algorithm) chooses only existing observations as centroids, which effectively minimizes the sum of distances within the cluster to the respective medoids. While the cluster centres using *k*-means can potentially lay outside the convex hull of a cluster, the *k*-medoids is considered more robust against outliers and noise. To overcome the randomness of the seed selection in *k*-means, using the centroids of an agglomerative clustering as initial seeds was proposed. This approach, called *k*-means++, therefore overcomes some of the stability issues introduced by the random seed selection. Within this work, we utilised hierarchical clustering, *k*-means and *k*-means++.

Cluster performance measures

Clustering differs from supervised ML approaches (where a model is trained on labelled data using a given algorithm and parameter setting) by not possessing a given ground truth. In supervised learning, a target variable, either a class (classification) or a numerical value (regression) is used to adjust the model during training and to assess the prediction on test data. In the case of classification, a number of common performance measures, such as predictive accuracy and receiver operator characteristics are available to assess the overall performance of the trained model. Similarly, in the regression case, measures such as the mean absolute error, root mean squared error or R^2 can be employed. In contrast, the performance of clustering analysis cannot be compared to any ground truth in general, and must be estimated using more indirect performance measures.

For real-world applications, no predefined labels or classes exist, so any performance measure for truly unsupervised machine learning employs some indication of how well the cluster algorithm has maximized intra-cluster similarity (high similarity within a cluster), while at the same time minimizing inter-cluster similarity (low similarity between examples in different clusters). In this study, the Silhouette Coefficient, Variance Ratio Criterion, Davies-Bouldin Criterion and Gap Statistic were employed in order to measure the consistency of the proposed cluster assignments.

<u>Silhouette coefficient</u>

The Silhouette Coefficient (SC) for each observation *i* is calculated as follows[6]:

$$sc(i) = \begin{cases} 1 - {a(i)}/{b(i)}, & if\ a(i) < b(i) \\ 0, & if\ a(i) = b(i) \\ {b(i)}/{a(i)} - 1, & if\ a(i) > b(i) \end{cases}$$

with $a(i)$ being the mean distance between i and all other observations within the same cluster:

$a(i) = \frac{1}{\lfloor C_i \rfloor - 1} \sum_{j \in C_i, j \neq i} d(i,j)$ (with $i \in C_i$ – observation i in cluster C_i)

and $b(i)$ being the mean distance between i and all observations in the nearest neighbouring cluster:

$b(i) = \min_{k \neq i} \frac{1}{\lfloor C_k \rfloor} \sum_{j \in C_k,} d(i,j)$ (with $j \in C_k$ – observation j in cluster C_k)

To assess the overall performance of a clustering result, the average of all coefficients $\tilde{s}(k) = \frac{\sum_{i \in D} sc(i)}{N}$ with k being the number of clusters and N the number of overall observations, is used. The closer $\tilde{s}(k)$ is to the theoretical maximum of 1, the better the average separation of each cluster to their respective neighbouring clusters. A value close to the theoretical minimum of -1 indicates an overlapping clustering. When establishing the best setting or algorithm for clustering, the maximum of the $\tilde{s}(k)$ should be sought ($argmax_{k \in \{1, \cdots, K\}}\ \tilde{s}(k)$).

The SC for the sinus rhythm cohort for 6 clusters using 9 embedding dimensions was 0.143, and for the AF cohort at 5 clusters and 9 embedding dimensions was 0.147.

Variance ratio criterion

The Variance Ratio Criterion (VRC), also called Calinski-Harabasz Criterion, measures the intra and inter-cluster variance of a clustering[7]. A high VRC indicates a clustering with better separation between clusters. The VRC is defined as:

$$VRC_k = \frac{SS_B}{SS_w} \times \frac{(N-k)}{(k-1)}$$

with N being the number of overall observations and SS_B (sum of squares *between* clusters) being the overall inter-cluster variance defined as:

$SS_B = \sum_{i=1}^{k} n_i \| m_i - m \|^2$

where k is the number of clusters, n_i is the number of observations in cluster i, m_i is the centroid of cluster i, m is the overall mean of the sample data and $\|m_i - m\|$ is the Euclidean distance between these two points (vectors).

The SS_W (sum of squares *within* clusters) being the overall intra-cluster variance defined as:

$SS_W = \sum_{i=1}^{k} \sum_{x \in c_i} \| x - m_i \|^2$ with x being an observation in cluster c_i and m_i being the centroid of the cluster.

The VRC for the sinus rhythm cohort for 6 clusters using 9 embedding dimensions was 2468.450 and for the AF cohort at 5 clusters and 9 embedding dimensions was 589.994.

Davies-Bouldin criterion

The Davies-Bouldin (DB) Criterion is measuring the ratio of within-cluster (intra) and between cluster distances (inter)[8], with a low score indicating better separation between clusters, calculated as:

$$\text{DB} = \frac{1}{k} \sum_{i=1}^{k} max_{j \neq i} \{D_{i,j}\}$$

where $D_{i,j}$ is the within-to-between cluster distance ratio for the ith and j^{th} clusters defined as:

$$D_{i,j} = \frac{(\bar{d_i} + \bar{d_j})}{d_{i,j}}$$

where $\bar{d_i}$ and $\bar{d_j}$ are the average distances of each observation in cluster i and j respectively and d_i, j is the distance between the centroids of the two clusters i and j.

The DB for the sinus rhythm cohort for 6 clusters using 9 embedding dimensions was 1.905, and for the AF cohort at 5 clusters and 9 embedding dimensions was 1.837.

Gap statistic

The gap statistic[9] compares the change in within-cluster dispersion with that expected under an appropriate reference null, or random, distribution. The reference null distribution is produced by clustering randomly generated data using the same features space (using a uniform distribution). The gap value is defined as:

$$gap_{d_k} = E_n^*\{log(W_k)\} - log(W_k) = \frac{1}{B}\sum_b log(W_{kb}^*) - log(W_k)$$

with the within-cluster dispersion W_k defined as

$$W_k = \sum_{r=1}^{k} \frac{1}{2n_r} D_r$$

and D_r being the sum of the pairwise distances for all points in cluster r and W_{kb}^* defined analogously for the sum of pairwise distances of the B random clusterings with $b \in \{1, \cdots, B\}$ and E_n^* denoting expectation under a sample of size n from the reference null distribution.

The gap value gap_{d_k} expresses the gap between the found clustering and the clusters from the randomly generated data for given number of clusters k.

The number of clusters k is chosen for the smallest k with the highest gap_{d_k} for which the following equation is satisfied:

$$d \in D: gap_{d_k} \geq gap_{d_{k+1}} - s_k$$

where s_k is defined as:

$$s_k = \sqrt{\left(\frac{1}{B}\sum_b log(W_{kb}^*) - \bar{l}\right)^2} \cdot \sqrt{1 + \frac{1}{B}}$$

with $\bar{l}$ as:

$$\bar{l} = \frac{1}{B}\sum_b log(W_{kb}^*)$$

The gap score for the sinus rhythm cohort for 6 clusters using 9 embedding dimensions was 1.654 and for the AF cohort at 5 clusters and 9 embedding dimensions was 1.617 (**Supplementary Figure 2**).

Evaluation

Jaccard Index

The Jaccard Index or Jaccard similarity coefficient j, is a statistic used to assess the similarity between two finite sets (A and B)[10]. It is defined as the size of the intersection divided by the size of the union of the two sets.

$$j = \frac{|A \cap B|}{|A \cup B|}$$

Overall, the Jaccard index can be used to express the similarity of using 1-j, the distance between two sets.

In clustering, the Jaccard index can be used to define the similarity of two clusterings agreement. For this, the average j is calculated for each label weighted by its support in the data.

Adjusted Rand Index

Similar to the Jaccard Index, the Rand Index (RI)[11] computes the similarity between two clusterings. For this it considers all pairs of samples and counts pairs that are assigned in the same cluster out of all possible pairs. The raw RI is defined as:

$$\text{RI} = \frac{|pairs\ agreeing|}{|all\ possble\ pairs|}$$

more mathematically:

Given a set of *n* elements

$$S = \{o_1, \cdots, o_n\}$$

and

$U = \{u_1, \cdots, u_R\}$ and $V = \{v_1, \cdots, v_C\}$

representing two partitions of S such that

$\bigcup_{i=1}^{R} u_i = S = \bigcup_{j=1}^{C} v_j$ and $u_i \cap u_{i'} = \emptyset = v_j \cap v_{j'}$ for $1 \leq i \neq i' \leq R$ and $1 \leq j \neq j' \leq C$.

Furthermore, let:

a, be number of pairs of elements in *S* that are in the same subset in *U* and in the same subset in *V*

b, be number of pairs of elements in *S* that are in different subsets in *U* and in different subsets in *V*

c, be number of pairs of elements in *S* that are in the same subset in *U* and in different subsets in *V*

d, be number of pairs of elements in *S* that are in different subsets in *U* and in the same subset in *V*

The RI is then defined as:

$$RI = \frac{a + b}{a + b + c + d}$$

The Adjusted Rand Index (ARI)[12] is the RI when adjusted for chance. It can be defined as:

$$ARI = \frac{RI - expected\ RI}{maximum\ RI - expected\ RI}$$

, which is bounded by 1 and -1, and takes on 0 when the index equals the expected value (i.e. when clustering is random).

Validation protocol

Using the generated cluster assignment in <data - study *x*> as the target, we trained random forest models to predict cluster membership for each leave-one-trial-out dataset. Subsequently, we predicted cluster membership for the excluded <study *x*> and compared this to the cluster assignment from the original clustering. The leave-one-study-out validation used the following approach:

- Given the complete cohort D_{all} and clustering C_{all}
- For each study $x \in Studies$ and data D_x:
  - use $D_{train} = D_{all} - D_x$ and $D_{test} = D_x$ with D_{all} represent the complete cohort and D_x the data for study x
  - cluster D_{train}, resulting in clustering C_{train}
  - use machine learning (i.e. random forests) to predict cluster membership of D_{test} using D_{train} as training data and C_{train} as target, resulting in C_{test}
  - Estimate a mapping function m_{train} able to map C_{train} to clusters C_{all} from D_{all} .
  - Estimate the performance using adjusted Rand index ARI_x on C_{all} and $m_{train}(C_{test})$ for all patients in D_{test}
- Report on weighted average of ARI for a $x \in Studies$.

Supplementary Table 1: Included and excluded trials

Trial acronym	Trial name	Participants	Summary of trial design if included, or reason for exclusion
**Included trials from the Beta-blockers in Heart Failure Collaborative Group (BB-meta-HF)**			
ANZ[13]	Australia/New Zealand Heart Failure Study	415	Randomised controlled trial of carvedilol versus placebo in patients with congestive heart failure due to ischaemic heart disease
BEST[14]	Beta-Blocker Evaluation Survival Trial	2707	Multicentre, randomized, double-blind trial of bucindolol versus placebo in patients with compensated congestive heart failure with LVEF ≤35%
CAPRICORN[15]	Carvedilol Post-Infarct Survival Control in LV Dysfunction Study	1959	Multicentre, randomised trial of carvedilol versus placebo in patients with a proven acute myocardial infarction and a left-ventricular ejection fraction of ≤40%
CIBIS-II[16]	Cardiac Insufficiency Bisoprolol Study II	2647	Multicentre double-blind randomised trial of bisoprolol versus placebo in symptomatic patients in NYHA class III/ IV, with left-ventricular ejection fraction of 35% or less receiving standard therapy with diuretics and inhibitors of angiotensin-converting enzyme.
COPERNICUS[17]	Carvedilol Prospective Randomized Cumulative Survival Study	2289	Multicentre, double-blind trial of carvedilol versus placebo in patients with more advanced heart failure (LVEF ≤25%)
MDC[18]	Metoprolol in Idiopathic Dilated Cardiomyopathy Study	383	Multicentre, randomised control trial of metoprolol versus placebo in patients with idiopathic dilated cardiomyopathy and LVEF <40%
MERIT-HF[19]	Metoprolol CR/XL Randomised Intervention Trial in Congestive Heart Failure	3991	Randomized, double-blind, control trial of metoprolol versus placebo in patients with symptomatic heart failure and LVEF ≤40%.
SENIORS[20]	Study of the Effects of Nebivolol Intervention on Outcomes and Rehospitalisation in Seniors with Heart Failure Study	2128	Randomised control trial of nebivolol versus placebo in patients ≥70 years old and a history of heart failure.
US-HF[21]	U.S. Carvedilol Heart Failure Study	1094	Double-blind, randomised trial of carvedilol versus placebo stratified by LVEF severity in patients with chronic heart failure
**Excluded trials from the Beta-blockers in Heart Failure Collaborative Group (BB-meta-HF)**			
CHRISTMAS[22]	Carvedilol Hibernating Reversible Ischaemia Trial: Marker of Success Study	383	Patients with atrial fibrillation were excluded in this trial
CIBIS-I[23]	Cardiac Insufficiency Bisoprolol Study	641	Creatinine level not recorded in this trial

LVEF = left ventricular ejection fraction; NYHA = New York Heart Association.

Supplementary Table 2: Baseline characteristics according to randomised treatment allocation

Characteristic	All patients N=15,659		Sinus rhythm N=12,822		Atrial fibrillation N=2,837	
	Placebo	Beta-blockers	Placebo	Beta-blockers	Placebo	Beta-blockers
Age, median years (IQR)	65 (55-72)	64 (55-72)	64 (54-72)	63 (54-71)	69 (60-74)	69 (60-74)
Women, n (%)	1794 (23%)	1914 (24%)	1543 (25%)	1642 (25%)	251 (18%)	272 (19%)
Body mass index, median kg/m^2 (IQR)	26.7 (24.1-29.8)	26.6 (24.0-29.7)	26.6 (24.0-29.7)	26.5 (23.9-29.7)	27.1 (24.4-30.3)	26.7 (24.1-29.7)
Heart rate, median beats/minute (IQR)	80 (70-88)	80 (72-89)	80 (72-88)	80 (72-88)	81 (72-92)	81 (73-91)
Systolic BP, median mmHg (IQR)	124 (110-140)	124 (110-140)	123 (110-140)	123 (110-138)	125 (113-140)	127 (113-140)
LVEF, median % (IQR)	27 (21-33)	27 (21-33)	27 (21-33)	27 (21-33)	27 (21-33)	27 (21-33)
Prior myocardial infarction, n (%)	4211 (55%)	4327 (54%)	3632 (58%)	3779 (58%)	549 (41%)	548 (39%)
NYHA class III/IV, n (%)	4336 (64%)	4466 (64%)	3452 (62%)	3596 (62%)	884 (73%)	870 (72%)
Creatinine, μmol/L median (IQR)	104 (88-124)	106 (88-125)	103 (88-124)	104 (88-124)	106 (90-129)	109 (90-132)
ACEi or ARB, n (%)	7349 (95%)	7528 (95%)	5988 (95%)	6200 (95%)	1361 (96%)	1328 (94%)
Any diuretic therapy, n (%)	6658 (86%)	6905 (87%)	5332 (85%)	5582 (85%)	1326 (93%)	1323 (94%)
Anticoagulation therapy, n (%)	2499 (32%)	2534 (32%)	1661 (26%)	1718 (26%)	838 (59%)	816 (58%)
Digoxin, n (%)	4530 (59%)	4769 (60%)	3332 (53%)	3587 (55%)	1198 (84%)	1182 (84%)

ACEi = angiotensin converting enzyme inhibitor; ARB = angiotensin receptor blocker; BP = blood pressure; IQR= interquartile range; LVEF = left-ventricular ejection fraction; NYHA = New York Heart Association.

Missing data report: not applicable as only participants with complete case data were used for analysis.

Supplementary Table 3: Cluster characteristics in sinus rhythm

Baseline characteristic	SR1 N=433	SR2 N= 1001	SR3 N= 1414	SR4 N=2537	SR5 N= 3497	SR6 N=3940
Age, median years (IQR)	48 (43-52)	66 (60-72)	69 (63-73)	70 (64-74)	52 (47-58)	66 (59-72)
Women, n (%)	31 (7%)	302 (30%)	886 (63%)	544 (21%)	684 (20%)	738 (19%)
Body mass index, median kg/m2 (IQR)	28.1 (26.7-30.9)	26.2 (24.1-29.1)	26.4 (23.6-29.4)	27.1 (24.8-29.9)	28.5 (25.7-32.2)	24.7 (22.5-27.2)
Heart rate, median beats/minute (IQR)	78 (72-85)	78 (68-80)	81 (74-89)	75 (69-81)	83 (75-92)	80 (73-90)
Systolic BP, median mmHg (IQR)	120 (110-130)	124 (112-135)	140 (127-152)	133 (122-147)	123 (110-138)	113 (104-124)
LVEF, median % (IQR)	32 (25-35)	35 (30-38)	31 (28-34)	33 (30-35)	24 (20-30)	21 (17-25)
Prior myocardial infarction, n (%)	352 (81%)	929 (93%)	448 (32%)	1877 (74%)	1407 (40%)	2397 (61%)
NYHA class III/IV, n (%)	59 (14%)	31 (3%)	1184 (84%)	975 (38%)	2936 (84%)	3292 (84%)
Creatinine, µmol/L median (IQR)	97 (84-110)	97 (80-110)	86 (73-99)	102 (88-120)	100 (86-118)	118(100-143)
ACEi or ARB, n (%)	419 (97%)	934 (93%)	1350 (95%)	2375 (94%)	3414 (98%)	3695 (94%)
Any diuretic therapy, n (%)	77 (18%)	52 (5%)	1343 (95%)	2325 (92%)	3304 (94%)	3812 (97%)
Anticoagulation therapy, n (%)	27 (6%)	93 (9%)	271 (19%)	396 (16%)	1074 (31%)	1518 (39%)
Digoxin, n (%)	39 (9%)	61 (6%)	929 (66%)	729 (29%)	2431 (70%)	2729 (69%)

ACEi = angiotensin converting enzyme inhibitor; ARB = angiotensin receptor blocker; BP = blood pressure; IQR, interquartile range; LVEF, left-ventricular ejection fraction; NYHA, New York Heart Association.

Supplementary Table 4: Validation protocol in the sinus rhythm cohort

Study	Number of participants		Adjusted Jaccard score		Adjusted Rand Index	
	Iteration set (all minus trial *x*)	Prediction set (study *x*)	Iteration set (all minus trial *x*)	Prediction set (study *x*)	Iteration set (all minus trial *x*)	Prediction set (study *x*)
CAPRICORN	11249	1573	0.589	0.471	0.533	0.459
US Carvedilol	12072	750	0.532	0.500	0.519	0.416
SENIORS	11693	1129	0.574	0.546	0.577	0.419
MERIT-HF	9625	3197	0.386	0.429	0.484	0.453
CIBIS II	10861	1961	0.708	0.692	0.608	0.591
ANZ	12468	354	0.598	0.372	0.610	0.393
MDC	12560	262	0.644	0.683	0.625	0.467
COPERNICUS	11393	1429	0.558	0.784	0.539	0.640
BEST	10655	2167	0.618	0.688	0.623	0.601
Average (95% CI)			0.579 (0.521 to 0.636)	0.574 (0.481 to 0.667)	0.569 (0.535 to 0.602)	0.493 (0.433 to 0.553)

Robustness of clustering and validity protocol in the 9 dimensions and 6 clusters model for all-cause mortality in sinus rhythm. Both the Jaccard score and Adjusted Rand Index were significantly different to random cluster assignment using repeated random forest models ($p<0.0001$ and $p=0.0198$, respectively).

Supplementary Table 5: Cluster characteristics in atrial fibrillation

Baseline characteristic	AF1 N=608	AF2 N=659	AF3 N=696	AF4 N=403	AF5 N=471
Age, median years (IQR)	74 (70-77)	58 (53-63)	73 (69-76)	71 (64-76)	61 (53-70)
Women, n (%)	38 (35%)	13 (16%)	28 (21%)	8 (5%)	8 (8%)
Body mass index, median kg/m2 (IQR)	27.0 (24.4-29.8)	29.1 (26.6-32.8)	26.3 (24.1-28.7)	25.6 (22.9-28.3)	25.8 (23.2-29.0)
Heart rate, median beats/minute (IQR)	80 (72-88)	84 (75-94)	80 (72-90)	80 (72-88)	88 (76-100)
Systolic BP, median mmHg (IQR)	135 (120-150)	130 (120-144)	132 (120-147)	113 (104-125)	110 (105-120)
LVEF, median % (IQR)	35 (32-38)	28 (23-33)	25 (21-30)	22 (18-26)	22 (18-26)
Prior myocardial infarction, n (%)	49 (45%)	11 (14%)	75 (55%)	111 (74%)	17 (17%)
NYHA class III/IV, n (%)	38 (35%)	69 (87%)	129 (94%)	136 (91%)	82 (79%)
Creatinine, µmol/L median (IQR)	96 (81-111)	95 (83-110)	110 (97-125)	156 (137-181)	115 (98-133)
ACEi or ARB, n (%)	102 (94%)	76 (96%)	128 (93%)	138 (92%)	102 (98%)
Any diuretic therapy, n (%)	88 (81%)	75 (95%)	133 (97%)	148 (99%)	101 (97%)
Anticoagulation therapy, n (%)	43 (39%)	44 (56%)	75 (55%)	108 (73%)	81 (78%)
Digoxin, n (%)	72 (66%)	71 (90%)	115 (84%)	131 (88%)	99 (95%)

ACEi = angiotensin converting enzyme inhibitor; ARB = angiotensin receptor blocker; BP = blood pressure; IQR, interquartile range; LVEF, left-ventricular ejection fraction; NYHA, New York Heart Association.

Supplementary Table 6: Validation protocol in the atrial fibrillation cohort

Trial (*x*)	Number of participants		Adjusted Jaccard score		Adjusted Rand Index	
	Iteration set (all minus trial *x*)	Prediction set (study *x*)	Iteration set (all minus trial *x*)	Prediction set (study *x*)	Iteration set (all minus trial *x*)	Prediction set (study *x*)
CAPRICORN	2701	136	0.658	0.887	0.585	0.928
US Carvedilol	2703	134	0.528	0.490	0.501	0.360
SENIORS	2337	500	0.592	0.828	0.493	0.873
MERIT-HF	2176	661	0.359	0.539	0.302	0.586
CIBIS II	2329	508	0.471	0.610	0.406	0.535
ANZ	2788	49	0.778	0.712	0.831	0.710
MDC	2787	50	0.646	0.337	0.637	0.073
COPERNICUS	2417	420	0.542	0.381	0.522	0.318
BEST	2458	379	0.718	0.521	0.683	0.400
Average (95% CI)			0.588 (0.504 to 0.672)	0.590 (0.466 to 0.713)	0.551 (0.449 to 0.653)	0.532 (0.351 to 0.712)

Robustness of clustering and validity protocol in the 9 dimensions and 5 clusters model for all-cause mortality in atrial fibrillation. Both the Jaccard score and Adjusted Rand Index were significantly different to random cluster assignment using repeated random forest models ($p<0.0001$ and $p=0.0264$, respectively).

Supplementary Figure 1: Simplified overview comparing neural networks with autoencoders

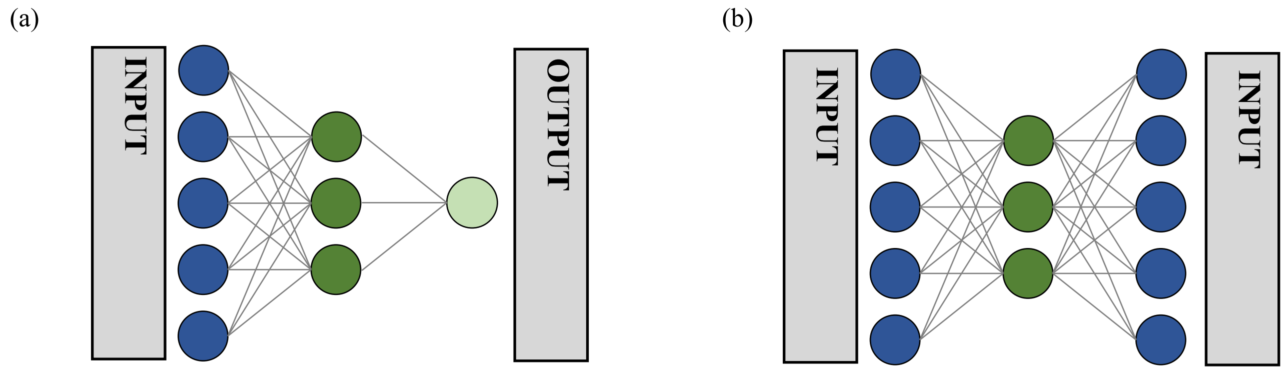

Simplified overview between (a) Neural Networks and (b) Autoencoders. Blue dots represent the input nodes and values; dark green dots represent the hidden layer(s); and the light green dot the output layer for prediction. In the case of (b), the autoencoder is trained to learn the identity function (it learns to reconstruct the input from the hidden layer, the so-called bottleneck layer).

Supplementary Figure 2: Cluster selection based on the gap statistic

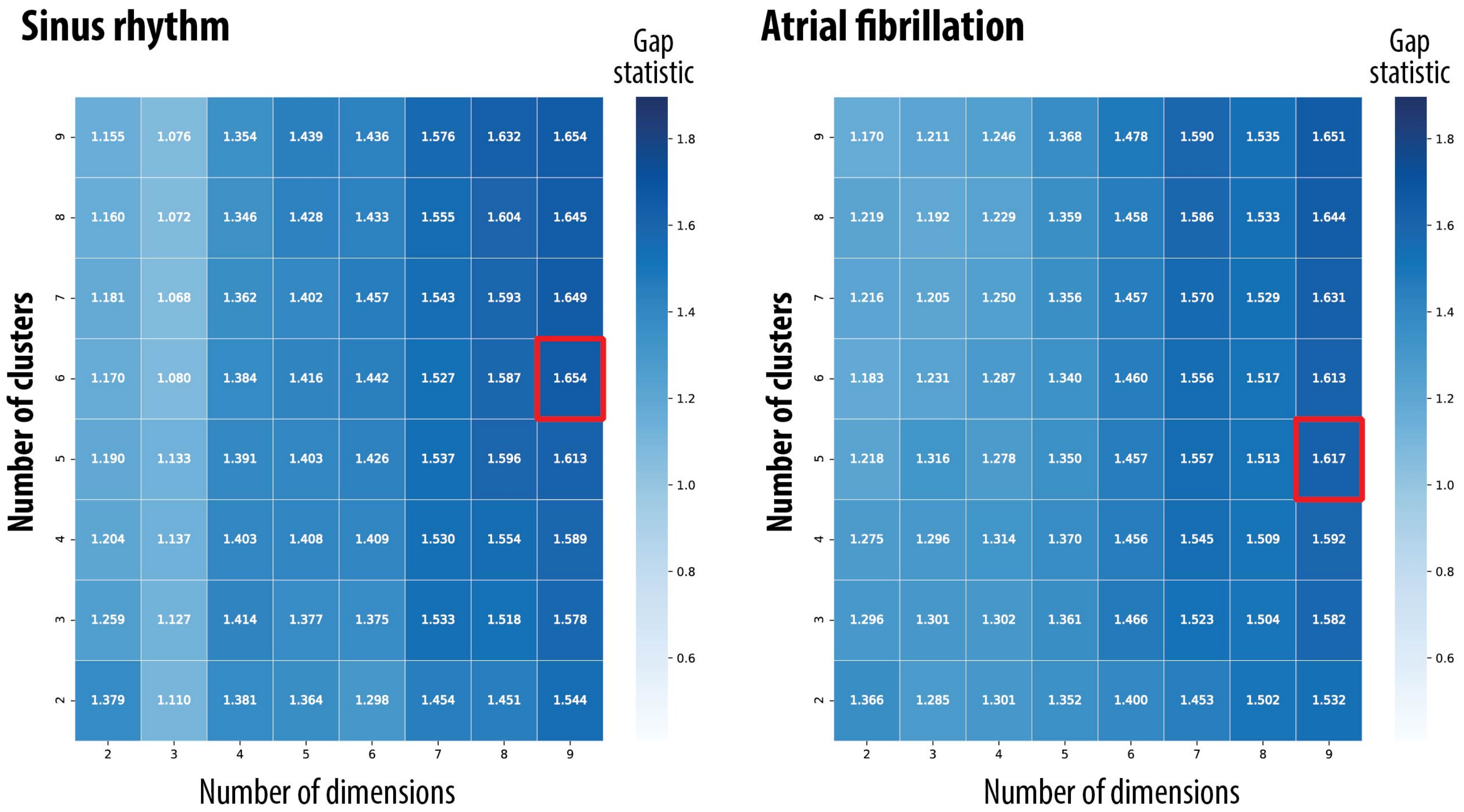

Heatmap plots of the gap values (gap statistics) for different embedding dimensions (x-axis) and the number of clusters (y-axis) for sinus rhythm and atrial fibrillation. The number of clusters for each analysis was objectively decided based on the gap statistic (higher better) with selection stopping for each dimension once a drop in the gap statistic was noted for an increasing cluster size. The final combination of dimensions and clusters for each cohort are indicated by red squares.

Supplementary Figure 3: Eight-cluster model for beta-blocker efficacy in sinus rhythm

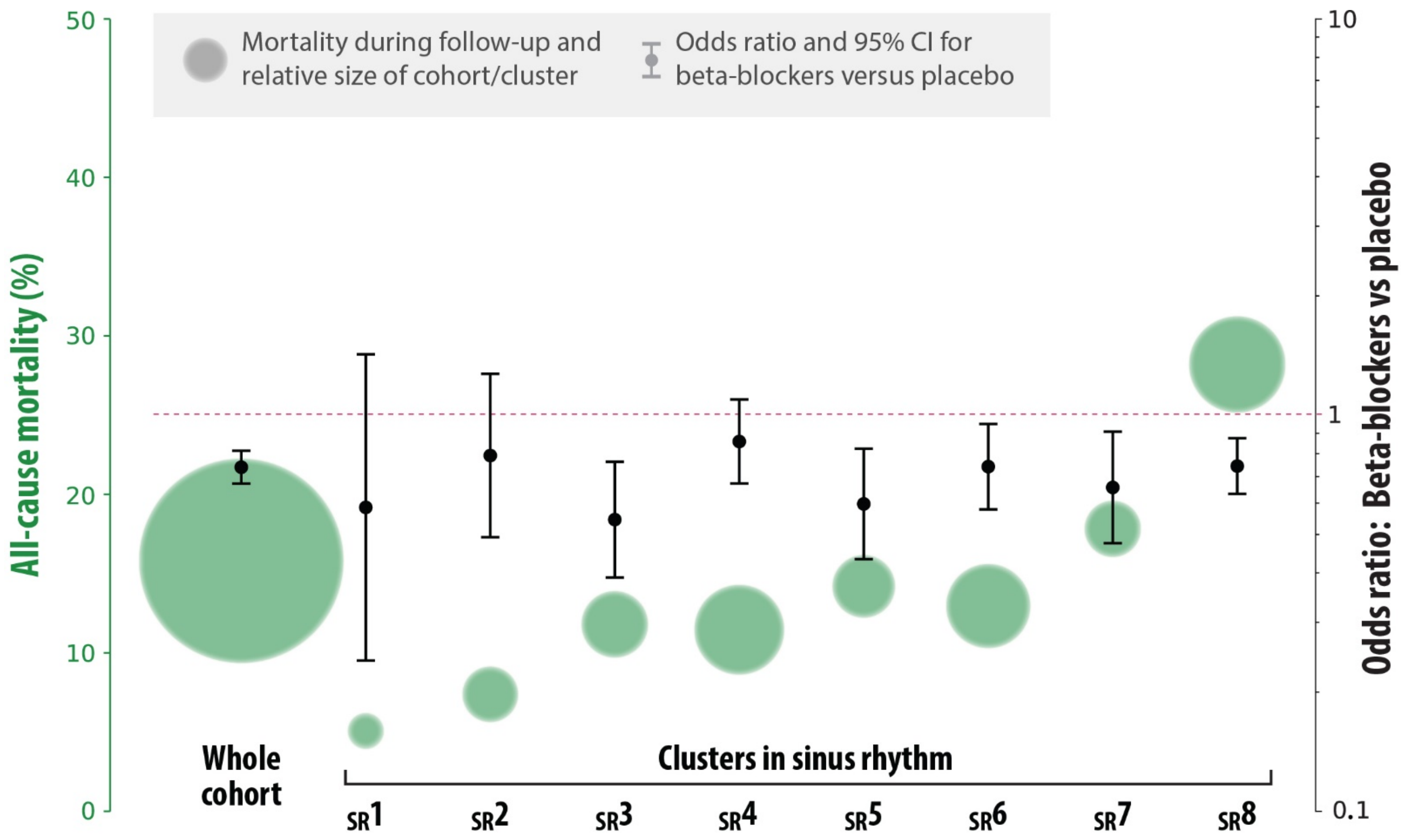

Green circles represent the average mortality risk, with size relative to the number of patients in that cluster. Odds ratios and associated 95% CI are for the efficacy of beta-blockers versus placebo for all-cause mortality in sinus rhythm (SR); odds below the red dotted line indicate a benefit from beta-blockers.

REFERENCES

1. Kramer MA. Nonlinear principal component analysis using autoassociative neural networks. *AIChE Journal* 1991; **37**(2): 233-43.

2. Hinton GE, Salakhutdinov RR. Reducing the Dimensionality of Data with Neural Networks. *Science* 2006; **313**(5786): 504-7.

3. Nazábal A, Olmos PM, Ghahramani Z, Valera I. Handling incomplete heterogeneous data using VAEs. *Pattern Recognit* 2020; **107**: 107501.

4. Ester M, Kriegel H-P, Sander J, Xu X. A density-based algorithm for discovering clusters in large spatial databases with noise. Proceedings of the Second International Conference on Knowledge Discovery and Data Mining. Portland, Oregon: AAAI Press; 1996. p. 226–31.

5. Kohonen T. Self-organized formation of topologically correct feature maps. *Biological Cybernetics* 1982; **43**(1): 59-69.

6. Rousseeuw PJ. Silhouettes: A graphical aid to the interpretation and validation of cluster analysis. *J Comput Appl Math* 1987; **20**: 53-65.

7. Caliński T, Harabasz J. A dendrite method for cluster analysis. *Comm Statistics* 1974; **3**(1): 1-27.

8. Davies DL, Bouldin DW. A Cluster Separation Measure. *IEEE Trans Pattern Anal Mach Intell* 1979; **PAMI-1**(2): 224-7.

9. Tibshirani R, Walther G, Hastie T. Estimating the number of clusters in a data set via the gap statistic. *J Royal Statistical Society: Series B (Statistical Methodology)* 2001; **63**(2): 411-23.

10. Jaccard P. The distribution of the flora in the alpine zone.1. *New Phytologist* 1912; **11**(2): 37-50.

11. Rand WM. Objective Criteria for the Evaluation of Clustering Methods. *J Am Statistical Assoc* 1971; **66**(336): 846-50.

12. Hubert L, Arabie P. Comparing partitions. *J Classification* 1985; **2**(1): 193-218.

13. Australia/New Zealand Heart Failure Research Collaborative Group. Randomised, placebo-controlled trial of carvedilol in patients with congestive heart failure due to ischaemic heart disease. *Lancet* 1997; **349**(9049): 375-80.

14. Beta-Blocker Evaluation of Survival Trial Investigators. A trial of the beta-blocker bucindolol in patients with advanced chronic heart failure. *N Engl J Med* 2001; **344**(22): 1659-67.

15. Dargie HJ. Effect of carvedilol on outcome after myocardial infarction in patients with left-ventricular dysfunction: the CAPRICORN randomised trial. *Lancet* 2001; **357**(9266): 1385-90.

16. The Cardiac Insufficiency Bisoprolol Study II (CIBIS-II): a randomised trial. *Lancet* 1999; **353**(9146): 9-13.

17. Packer M, Coats AJ, Fowler MB, et al. Effect of carvedilol on survival in severe chronic heart failure. *N Engl J Med* 2001; **344**(22): 1651-8.

18. Waagstein F, Bristow MR, Swedberg K, et al. Beneficial effects of metoprolol in idiopathic dilated cardiomyopathy. Metoprolol in Dilated Cardiomyopathy (MDC) Trial Study Group. *Lancet* 1993; **342**(8885): 1441-6.

19. Effect of metoprolol CR/XL in chronic heart failure: Metoprolol CR/XL Randomised Intervention Trial in Congestive Heart Failure (MERIT-HF). *Lancet* 1999; **353**(9169): 2001-7.

20. Flather MD, Shibata MC, Coats AJ, et al. Randomized trial to determine the effect of nebivolol on mortality and cardiovascular hospital admission in elderly patients with heart failure (SENIORS). *Eur Heart J* 2005; **26**(3): 215-25.

21. Packer M, Bristow MR, Cohn JN, et al. The effect of carvedilol on morbidity and mortality in patients with chronic heart failure. U.S. Carvedilol Heart Failure Study Group. *N Engl J Med* 1996; **334**(21): 1349-55.

22. Cleland JG, Pennell DJ, Ray SG, et al. Myocardial viability as a determinant of the ejection fraction response to carvedilol in patients with heart failure (CHRISTMAS trial): randomised controlled trial. *Lancet* 2003; **362**(9377): 14-21.

23. CIBIS Investigators and Committees. A randomized trial of beta-blockade in heart failure. The Cardiac Insufficiency Bisoprolol Study (CIBIS). *Circulation* 1994; **90**(4): 1765-73.
